# Looking for ideas: Eye behavior during goal-directed internally focused cognition☆

**DOI:** 10.1016/j.concog.2017.06.009

**Published:** 2017-07-06

**Authors:** Sonja Walcher, Christof Körner, Mathias Benedek

**Affiliations:** Institute of Psychology, University of Graz, Universitätsplatz 2, 8010 Graz, Austria

**Keywords:** Self-generated thought, Perceptual decoupling, Eye movements, Idea generation, Internally directed cognition

## Abstract

Humans have a highly developed visual system, yet we spend a high proportion of our time awake ignoring the visual world and attending to our own thoughts. The present study examined eye movement characteristics of goal-directed internally focused cognition. Deliberate internally focused cognition was induced by an idea generation task. A letter-by-letter reading task served as external task. Idea generation (vs. reading) was associated with more and longer blinks and fewer microsaccades indicating an attenuation of visual input. Idea generation was further associated with more and shorter fixations, more saccades and saccades with higher amplitudes as well as heightened stimulus-independent variation of eye vergence. The latter results suggest a coupling of eye behavior to internally generated information and associated cognitive processes, i.e. searching for ideas. Our results support eye behavior patterns as indicators of goal-directed internally focused cognition through mechanisms of attenuation of visual input and coupling of eye behavior to internally generated information.

## 1 Introduction

The peculiar gaze of someone deeply absorbed in thought or “staring into space” suggests that eye behavior during internally focused attention might be different from states of externally focused attention. In fact, recent research provides evidence that mind wandering, the involuntary slipping away of attention from an external task to an unrelated internal train of thought, is associated with characteristic changes in eye behavior such as spontaneous pupil activity (Franklin, Broadway, Mrazek, Smallwood, & Schooler, 2013; Smallwood et al., 2011, 2012). Besides this spontaneous form of internally focused cognition, there are also many goal-directed cognitive activities which require a voluntary shift of attention to an internal focus such as planning or idea generation. For such activities external visual information is irrelevant and can even be distracting. Eye behavior is assumed to differ between states of goal-directed internally and externally focused cognition. This study aims to examine the characteristic eye behavior during goaldirected internally focused cognition.

“Internally directed cognition” or stimulus-independent thought (Smallwood & Schooler, 2006) refers to cognition with an attentional focus on internally generated information rather than the external environment. Internally generated information results from memory retrieval of internal representations and combinations and modifications of those representations (mental simulations, ideas, dreams; see Chun, Golomb, & Turk-Browne, 2011; Dixon, Fox, & Christoff, 2014). The literature suggests that eye behavior may respond to and even support internal cognition in at least two ways: first, by means of attenuation of potentially distracting perceptual input, and second, by means of a coupling of eye behavior to internally generated information and processes. We will briefly review evidence in support of these two hypothesized mechanisms.

### 1.1 Attenuation of visual input

Due to limited information processing capacity, processing external and internal information simultaneously impairs performance in one domain or both (Chun et al., 2011; Dixon et al., 2014). On the one hand, external tasks are impaired by internal events, like daydreaming or trying to solve a puzzle whilst driving a car (He, Becic, Lee, & McCarley, 2011; Savage, Potter, & Tatler, 2013). On the other hand, tasks requiring internally focused cognition, like thinking about an amazing new idea and how to implement it, can get interrupted by more or less irrelevant external events, such as an e-mail popping up on one’s desktop. External (perceptual input) and internal (self-generated) information are competing for limited processing resources (Chun et al., 2011). Therefore, being able to focus on one’s internal stream of thought and effectively shielding it from distracting external information has a high influence on the resulting performance. Active attenuation or shut out of external information can occur at different levels of visual perception.

At the neural level, research using EEG alpha frequency activity and functional MRI suggests an active suppression of visual input when participants have to solve a divergent thinking task with high internal processing demands. For example, EEG alpha activity is assumed to reflect a top-down mechanism for suppression of external visual information processing (Benedek, Bergner, Könen, Fink, & Neubauer, 2011; Benedek, Schickel, Jauk, Fink, & Neubauer, 2014; Fink & Benedek, 2013). Moreover, the right inferior parietal cortex was implicated in the down-modulation of visual information processing during high internal attention demands (Benedek et al., 2016; for an overview, see Benedek, 2017).

At the level of eye behavior, the most straightforward mechanism would be to simply look away from distracting stimuli (e.g. avoid eye contact: gaze aversion) or close the eyes. Indeed, gaze aversion and eye closure were frequently observed during demanding internally focused cognition and are thought to reduce cognitive load and thereby free up cognitive resources (Doherty-Sneddon & Phelps, 2005; Mastroberardino & Vredeveldt, 2014; Vredeveldt, Hitch, & Baddeley, 2011). The frequency of gaze aversion depends on cognitive load. The more resources an internally focused task demands, the more resources need to be recruited/withdrawn elsewhere, e.g. via gaze aversion or eye closure, to prevent performance impairments (Doherty-Sneddon & Phelps, 2005).

Very short eye closures – blinks – may also reduce the processing of visual input and enhance internally focused cognition. Solving problems through spontaneous insight was associated with higher blink rates as compared to analytical problem solving (Salvi, Bricolo, Franconeri, Kounios, & Beeman, 2015). The relationship between mind wandering and blink rate is currently not clear (Mooneyham & Schooler, 2016; Smilek, Carriere, & Cheyne, 2010; Uzzaman & Joordens, 2011), but demanding internally focused cognition tasks like idea generation and insight problem solving are typically accompanied by higher blink rates (Akbari Chermahini & Hommel, 2012; Salvi et al., 2015; Ueda, Tominaga, Kajimura, & Nomura, 2015), supporting the hypothesis of an active decoupling strategy.

Besides actual interruption of the visual input via blinks or gaze aversion, the processing of external visual information can also be attenuated. One potential oculomotor mechanism for attenuation would be disaccommodation. To focus on an object in three-dimensional space the eyes need to be aligned (eye vergence) and the lens adjusted to the object’s distance. These changes are associated with changes in pupil diameter. The three processes – eye vergence, lens constriction and pupil size changes – are coupled in the so-called convergence reflex or near response triad (Delank & Gehlen, 2006; McLin & Schor, 1988; Myers & Stark, 1990). Defocusing an object results in blurring and double vision, impairing further processing of visual information. Therefore, eye vergence is an important variable in research on covert attention - when attention is shifted to another location without moving the eyes (Solé Puig, Pérez Zapata, Aznar-Casanova, & Supèr, 2013) - and dyslexia (e.g. Kapoula et al., 2007), and it could also play a role in goal-directed internally focused cognition as suggested by the phenomenon of “staring into space”. The phenomenon of “staring into space” refers to the peculiar gaze people have when they are deeply absorbed in thought. “Staring into space” seems to be characterized by a strong stare (lack of eye movements) and the eyes seem to focus at a distance far away.

In a similar way, attenuation of visual perception could also be achieved by means of reduced microsaccade activity. When fixating static stimuli, neuronal adaptation leads to perceptual fading within seconds. To counteract perceptual fading, our eyes perform fixational eye movements (microsaccades, drift and tremor), of which microsaccades are considered the most important ones (Martinez-Conde, Macknik, Troncoso, & Dyar, 2006; Martinez-Conde, Otero-Millan, & Macknik, 2013; McCamy et al., 2012). Microsaccades cannot be generated voluntarily, but they can be suppressed voluntarily for a few seconds (Bridgeman & Palca, 1980; Winterson & Collewun, 1976). As external visual information is not needed during periods of internally focused cognition, there is no need to counteract fading through microsaccades. Fading may even facilitate internally focused cognition by reducing distracting visual input. So far, the role of eye vergence and microsaccades for internally focused cognition has not been addressed by research.

### 1.2 Coupling to internally generated information

When we remember a special occasion or think about the upcoming holiday, we often seem to actually look at a mental picture in our mind’s eye. This notion has been supported by research on mental imagery and memory retrieval, showing that eye movements are commonly coupled to cognitive processes during internally focused cognition (Ferreira, Apel, & Henderson, 2008; Johansson, Holsanova, Dewhurst, & Holmqvist, 2012; Johansson, Holsanova, & Holmqvist, 2005). When retrieving information about objects from memory, eye behavior patterns reflect those made while actually looking at the scene (Johansson et al., 2005). Even when eye movements during encoding are prohibited through continuous fixation of a certain point, eye movement patterns during retrieval reflect spatial characteristics of the remembered material, as one was looking at it. Those eye movements during retrieval seem to play an important role for retrieval itself, as prohibiting them impairs retrieval (Johansson et al., 2012).

Furthermore, irrelevant eye movements impair spatial imagery (e.g. navigating through a matrix) but not visual mental imagery (e.g. imagining features of objects, for instance whether the letter A contains a curve or not), suggesting that eye movements are more crucial for spatial than depictive aspects of content (de Vito, Buonocore, Bonnefon, & Della Sala, 2014). It is even possible to determine whether a visual or a linguistic task was performed using eye movement patterns although the presented stimuli were the same pictures (Coco & Keller, 2014). Also searching for and focusing on information in long-term memory is reflected in different eye movement patterns. These differences have a counterpart in viewing external visual information: there are more eye movements during search and only a few when focusing on an object (Ehrlichman & Micic, 2012).

Cognitive processes do not only affect fixations and saccades, but also pupil diameter. Pupil diameter adjusts to imagined luminance changes (Laeng & Sulutvedt, 2014), and even responds to other properties of the imagined content such as valence, cognitive load or target detection (Palinko, Kun, Shyrokov, & Heeman, 2010; Piquado, Isaacowitz, & Wingfield, 2010; Privitera, Renninger, Carney, Klein, & Aguilar, 2010). Further evidence comes from studies on mind wandering, showing that moving attention away from external events results in spontaneous, more variable pupil activity (Franklin et al., 2013; Smallwood et al., 2011). Moreover, mind wandering episodes while reading reflected deviations from typical reading behavior including longer fixation duration and reduced within-word regressions (Reichle, Reineberg, & Schooler, 2010; Uzzaman & Joordens, 2011).

### 1.3 The present study

The presented literature suggests that internally focused cognition is accompanied by characteristic eye behavior changes supporting the decoupling from external information and coupling to internally generated information. Much of this evidence comes from studies examining differences between goal-directed externally focused cognition (e.g., reading) and spontaneous, internally focused cognition (i.e., mind wandering). Additionally, previous studies often focused on subsets of specific oculometric parameters (e.g., blinks or pupil diameter) and their role for either decoupling or coupling of eye behavior to internal processes. Therefore, the interplay of various oculometric parameters and their integrative functional role for perceptual decoupling and coupling mechanisms during internally focused cognition still need to be investigated.

The present study aims to extend available research in at least two important ways: first, we aim to identify specific eye behavior patterns associated with deliberate internally focused cognition by contrasting two goal-directed thinking tasks, one with an external focus (letter reading) and one with an internal focus of attention (idea generation). Second, we consider a large range of oculometric parameters including less common measures like eye vergence and microsaccade rate that appear particularly relevant to examine potential mechanisms of perceptual decoupling. The broad range of oculometric parameters allows us to get an integrated picture of eye behavior during internally focused cognition and the interplay of both mechanisms: attenuation of visual input and coupling to internal processes. Finally, we explore the robustness of all effects by means of an experimental variation of luminance across trials.

The *attenuation of visual input* hypothesis predicts that goal-directed internally focused cognition should be associated with higher blink rates and blink durations to reduce visual input (Salvi et al., 2015). Additionally, we explore the role of eye vergence and microsaccade activity as a more nuanced mechanism to attenuate visual input. Internally focused cognition may be related to a smaller angle of eye vergence (indicating a focus behind the screen) and to fewer microsaccades, counteracting perceptual fading.

Predictions regarding the *coupling to internally generated information* hypothesis are less straightforward. Our external task requires only little eye movements for reading single letters in the center of the screen. Therefore, we expect that searching for an idea – similar to searching for an object in a visual environment (Ehrlichman & Micic, 2012) – is reflected in a more active eye behavior. This eye behavior could manifest itself in more and shorter fixations, more saccades and saccades with greater amplitude (Ehrlichman & Micic, 2012). Pupil diameter (Laeng & Sulutvedt, 2014) and eye vergence (McLin & Schor, 1988) also respond to imagined differences in luminance and distance, respectively. Imagined objects and their uses during the idea generation may vary in luminance and distance within one trial. Therefore, we expect more within-trial variation of pupil diameter (Smallwood et al., 2011) and eye vergence.

## 2 Method

### 2.1 Participants

Forty-eight young adults (23 ± 4 years old, 32 females, 15 males, 1 non-binary gender identity), mostly university students, participated in the experiment for course credit and/or the possibility to win a skiing holiday. All participants had normal or corrected-to-normal (soft contact lenses) vision, reported no strabismus or other medical condition affecting vision. All participants gave written informed consent. Two additional participants had to be excluded from analyses. One had excessive missing data (> 50%) due to partial occlusion of the pupil by the eye lids, the other had slight strabismus distorting gaze data. The study was approved by the local ethics committee.

### 2.2 Stimuli and apparatus

**Fig. 1:**
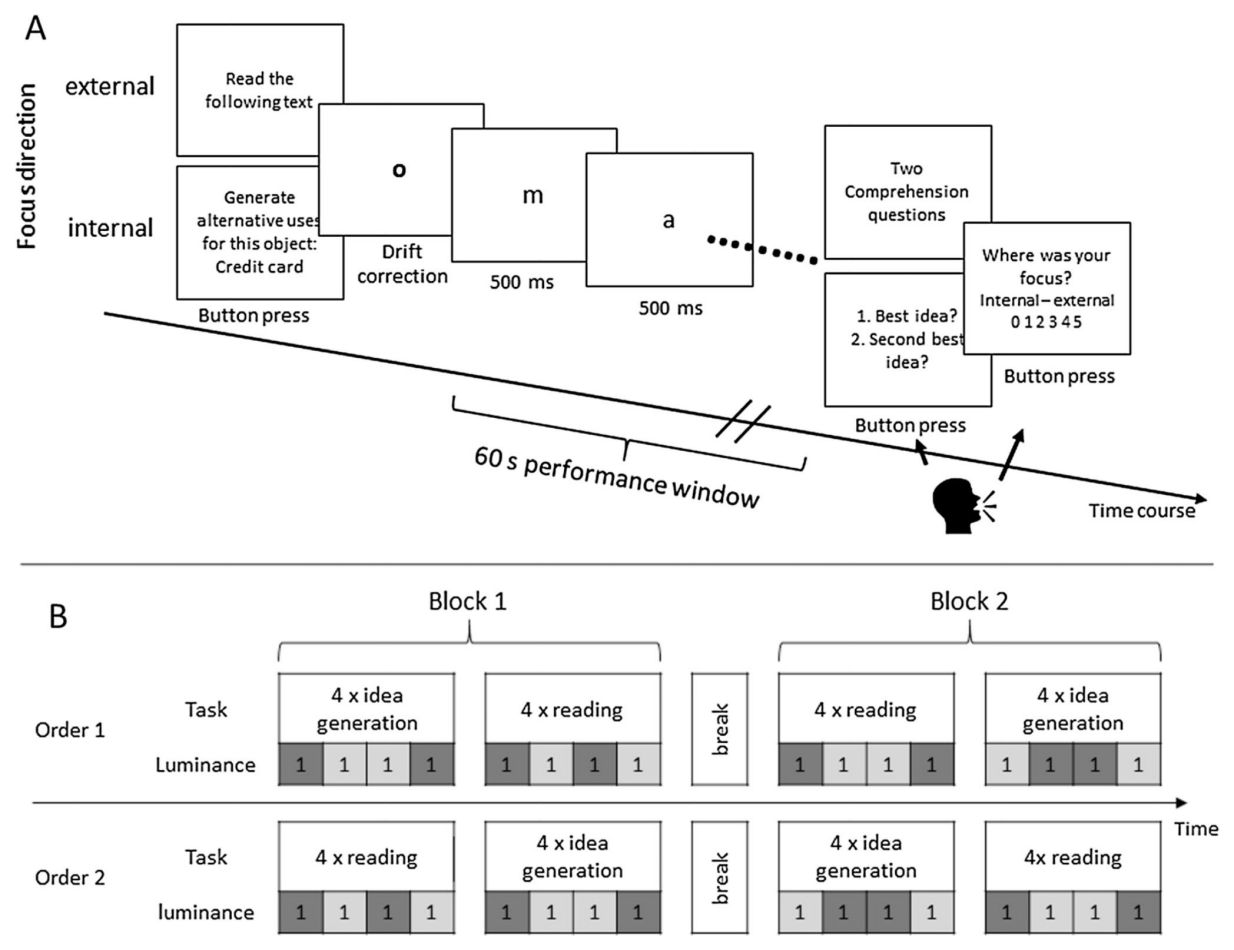
(A) Sequence of events in a trial for the external (reading) and internal (idea generation) task. (B) Sequence of tasks and luminance conditions. Half of the participants performed the tasks in order 1, the other half in order 2.

As stimuli, a stream of letters was presented in the center of the screen. All letters were lowercase, and presented in black Arial font of size 15 pt resulting in a character width of 0.32° visual angle (ca. 2.5 mm or 8.5 pixels). Umlauts were written-out (e.g. “ä” to “a – e”) and words were separated by a dash (“-”) instead of a space so the screen was never empty (see Fig. 1A). Letters were presented for 500 ms each, directly followed by the next letter. To test possible luminance effects on eye parameters, background luminance was brighter (RGB color code: 204,204,204) in one half of the trials and darker (102,102,102) in the other half.

Participants were placed in a sound attenuated room with the lights turned on and sat at a distance of 50 cm from the screen. Their heads were stabilized using chin rest and forehead rest of the EyeLink Tower Mount (SR Research, Ontario, Canada). Stimuli were presented on a 19-in LG flatroon L1920P monitor run at 60 Hz and a 1240 × 1024 pixels resolution, subtending 29.4 pixels per degree visual angle.

Binocular eye data were recorded using an EyeLink 1000 Plus Tower Mount eye tracker (SR Research, Ontario, Canada) with a temporal resolution of 500 Hz. For stimulus presentation and response recording, the EyeLink Experiment Builder software (SR Research, Ontario, Canada) was used. For calibration, validation, drift correction and computation of the eye movement parameters (blinks, fixations, saccades), we used the manufacturer’s software (SR Research, Ontario, Canada). Online velocity threshold for saccade detection was set to 35°/s and acceleration threshold to 9500°/s^2^. There was a 9-point calibration procedure before each block and a drift correction before each trial. Spatial resolution was typically better than 0.30°. Participants’ answers were recorded with a microphone to monitor and record task performance.

### 2.3 Procedure and task

Participants performed eight experimental trials of a reading task and eight trials of an idea generation task (see Sections 2.3.1 and 2.3.2), preceded by one practice trial of each task. The experimental trials were organized in two blocks with a break in between to minimize effects of visual fatigue. Each block comprised four consecutive reading trials and four consecutive idea generation trials (half with dark and half with bright background, respectively). Half of the participants started with the reading task, the other half with the idea generation task (see Fig. 1B). After each block, participants filled out a short questionnaire regarding aspects of task performance, including questions about their concentration, the perceived demands of tasks and the amount of distraction by the letter stream. Between blocks participants also filled out personality questionnaires (part of another study) in the antechamber and were instructed to take as much time for the break as they needed to rest their eyes (at least 10 min). The experiment took about 45 min in total.

#### 2.3.1 Externally focused cognition: Reading task

A reading task was used as externally focused cognition task. Participants were required to read a German text of 120-character length. To reduce saccades, the text was presented as a stream of consecutive letters, each presented for 500 ms at the middle of the screen, resulting in a presentation duration of 60 s for the entire text (see Fig. 1A). After text reading, two comprehension questions on the content of the message were presented on the screen to test if participants had focused on the letters throughout the task (e.g., text: “Albert and I are going to a concert on Tuesday. I asked Franz if he wants to join us, but he visits his grandmother that day” Questions: “What day are we going to the concert?”, “Who is Franz visiting?”). Short mind-wandering episodes during this task would lead to problems in understanding the text and in answering the questions. In a third question, participants had to indicate the direction of attentional focus during the task, from 0 = “totally absorbed in thought” to 5 = “totally focused on external events”. Participants were informed that if they were focusing on the letters of the text during the whole trial they should indicate 5. If they had trouble to concentrate on the letters and were mind wandering during the whole trial, they should indicate 0. Participants answered the questions aloud. At the end of each trial participants pressed the spacebar to continue with the next trial.

#### 2.3.2 Internally focused cognition: Idea generation task

As an internally focused cognition task, we used the alternate uses task (Guilford, 1967), a popular and well-established idea generation task commonly employed in research on divergent thinking and creativity (Kaufman, Plucker, & Baer, 2008). The alternate uses task asks to generate creative uses for a common household object (e.g., paper cup). This task relies on imagination based on an initially presented stimulus word and was found to be independent of further sensory processing (Benedek et al., 2014).

The procedure of the idea generation task was essentially the same as in the reading task (see Fig. 1A): at the beginning of each trial participants received the instruction (e.g., “find as many and as creative alternative uses for this object: credit card”). Then they had 60 s to think of creative uses but without verbalizing them. During this period, the same letters as in the reading task were presented one character at a time, but in reverse order to make it unreadable. At the end of each task, participants were prompted to tell their two best ideas. Finally, participants had to indicate again their attentional focus during the task. They were informed that if they were focusing on idea generation during the whole trial, they should indicate 0 = “totally absorbed in thought”. If they were constantly distracted by the letters on the screen or any other external events and could not focus on their ideas, they should indicate 5 = “totally focused on external events”.

### 2.4 Data analysis

Creativity of the generated ideas was rated by four experienced raters (overall inter-rater reliability of 0.67) on a four-point scale ranging from 0 = “not creative” to 3 = “very creative” (Diedrich, Benedek, Jauk, & Neubauer, 2015; Silvia et al., 2008). Answers to the comprehension questions after each reading task trial were rated as correct or incorrect and percentage of correct answers was analyzed.

Regarding eye tracking data, fixation durations and counts of fixations, blinks and saccades and saccade amplitudes per trial were calculated offline using Data Viewer software (SR Research, Ontario, Canada). Based on the default settings of the Data Viewer (SR Research, Ontario, Canada) saccades were defined as eye movements that exceeded 30°/sec velocity, 8000°/sec^2^ acceleration and/or 0.15° motion. Blinks were defined as a period with the pupil data missing for three or more samples in a sequence (= at least 6 ms) and fixations were defined as any period that is not a blink or a saccade.

Blinks as well as additional 200 ms periods before and after each blink were removed from gaze position and pupil diameter data to eliminate parts where the pupil was partially occluded (McCamy et al., 2012).

Only data for which the eye tracker had recorded both eyes were analyzed. Further data analyses were performed using R (www.r-project.org). For calculation of pupil diameter and eye vergence, eye tracking data were down-sampled from 500 Hz to 50 Hz by averaging across 10 data points (20 ms). Pupil diameter data were transformed from arbitrary units (measured by the eye tracking software) to z-values to make them comparable across subjects.

Calculation of angle of eye vergence was similar to methods applied in previous research (Solé Puig et al., 2015, 2013). Using participants’ individual inter-pupil distance (measured with a transparent ruler), gaze positions of both eyes in mm and distance between observer and screen (50 cm), gaze vectors for each eye were calculated. Gaze position coordinates were transformed from pixels to mm (3.4 pixels per mm). Gaze positions with fixation disparities outside the margins of participants’ pupil distance plus 10 mm in both sides (negative and positive fixation disparity) were removed as artefacts, as fixation disparities of this size do not occur during normal gaze behavior of healthy adults. The intersection point (or closest approximation if vectors did not intersect) of the right and left gaze vectors was calculated with the function qr.solve of the {base} R-package (www.r-project.org). The distance of the intersection point from midpoint between eyes was used as length of gaze vector. Angle of eye vergence in degrees was then calculated with the following formula:

$$\text{Angle}\;\text{of}\;\text{eye}\;\text{vergence}=2*\text{atan}\left(\frac{\text{mean}\;\text{pupil}\;\text{distance}/2}{\text{length}\;\text{of}\;\text{gaze}\;\text{vector}}\right)*\frac{180}{\pi }$$

For the mean pupil distance a length of 60 mm was used for all participants.

For microsaccade calculation, original 500 Hz gaze position data were used. Microsaccades (count and amplitude) were determined using the Microsaccade Toolbox for R (Engbert, Sinn, Mergenthaler, & Trukenbrod, 2015) with *λ* = 4 and a minimum microsaccade duration of 6 ms. Microsaccades were defined as saccades with an amplitude smaller than 1.0° (McCamy et al., 2012). Only binocular microsaccades with a minimum overlap of one data sample were considered. Microsaccade amplitude was defined as the mean across both eyes.

For all eye parameters, means or within-trial variability was computed for each trial. Trials with more than 50% missing data and trials with values beyond three standard deviations from the individual’s mean were discarded (percent trials discarded: *M* = 3.78, *SD* = 5.58, Max = 25.0). Remaining trials were averaged for the reading and the idea generation trials and for the bright and darker background luminance, respectively. For raw and processed data as well as additional analysis see Walcher, Körner, and Benedek (2017).

## 3 Results

### 3.1 Task performance

On average, participants were able to correctly answer 90.36% of the comprehension questions in the reading task. In the idea generation task, participants reported two ideas in 98.31% of trials. All trials were included in further analysis.

Participants reported being more focused on external events during the reading task than during the idea generation task (reading: *M* = 3.20, *SD* = 0.84, idea generation: *M* = 2.27, *SD* = 1.02, *t*(47) = 5.23, *p* < .001, *r* = .61). There were no task differences between ratings on how well they could concentrate (reading: *M* = 3.03, *SD* = 1.20, idea generation: *M* = 3.01, *SD* = 1.21, *t*(47) = 0.10, *p* = .925, *r* = .01). The letters were perceived as relatively little distracting during the idea generation task (*M* = 1.72, *SD* = 1.39, on a scale from 0 = *not distracting at all* to 5 = *totally distracting*). Participants reported that they perceived the idea generation task as more demanding than the reading task (reading: *M* = 1.91, *SD* = 1.24, idea generation: *M* = 2.58, *SD* = 1.13, *t* (47) = 3.95, *p* < .001, *r* = .50).

### 3.2 Eye tracking data

A total of 12 eye movement variables were analyzed, which are not necessarily independent from each other (e.g. blink count correlated with fixation count up to *r*_S_ = .58, *p* < .001 and with saccade count up to *r*_S_ = .58, *p* < .001). Most of the eye movement variables violated the assumption of a normal distribution on the sample level (see Table 1, asterisk indicates a significant Shapiro-Wilk test). Medians for all tasks and conditions are reported in Table 1. Univariate ANOVAs indicated no interaction effects of task type (idea generation, reading) and background luminance on eye parameters (bright, dark; all *p*s > .05). Hence, effects of task type (idea generation versus reading) and background luminance were analyzed separately using non-parametric Wilcoxon rank-sum tests (for calculation of effect size *r* see Field, 2013).

#### 3.2.1 Effects of reading vs. idea generation

**Fig. 2:**
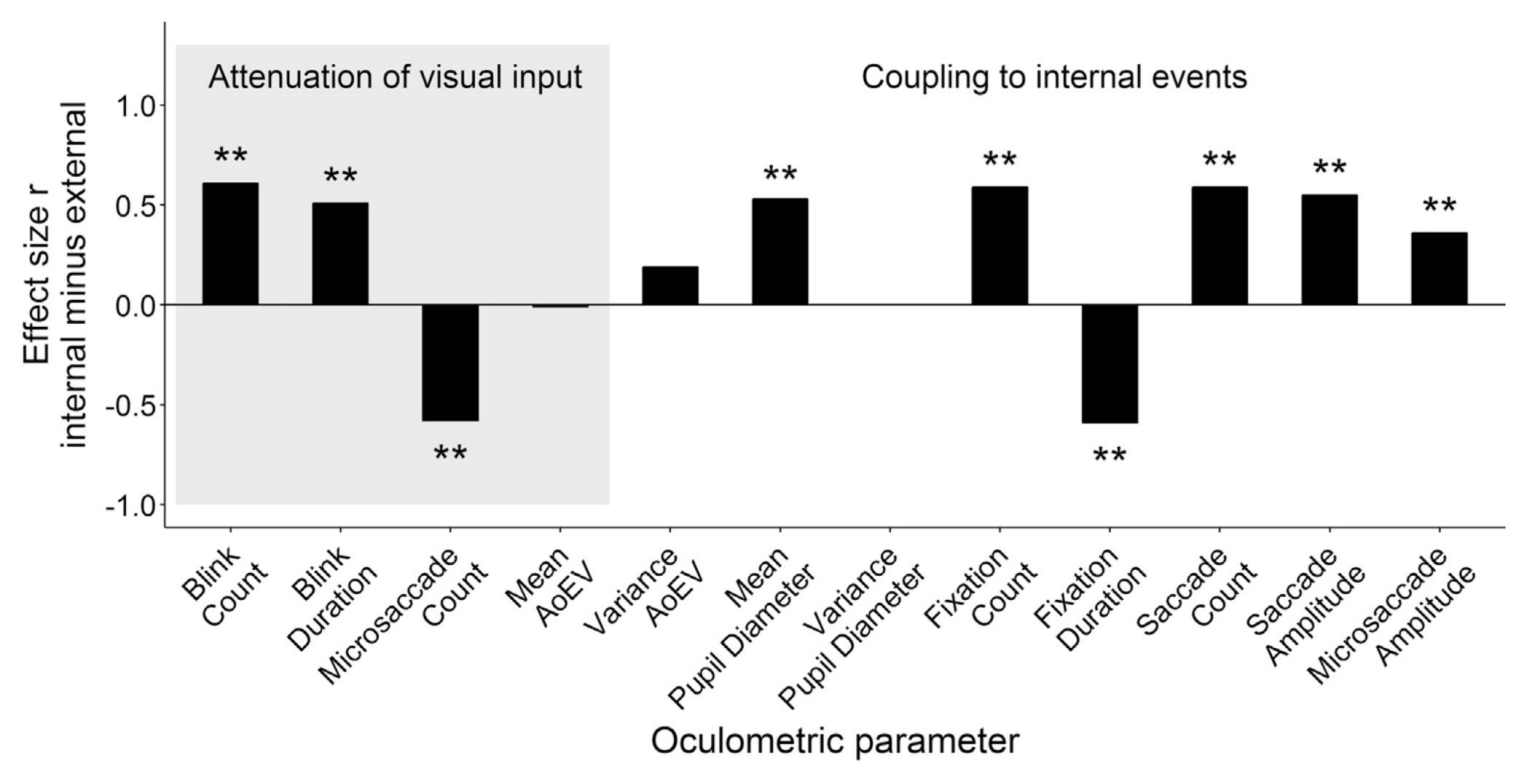
Effects of internally and externally focused cognition on oculometric parameters illustrated by effect size r. Positive effect sizes indicate higher values of the oculometric parameter in the internal compared to the external task. Effects presumably associated with attenuation of visual input are shaded; effects presumably associated with coupling to internal events are unshaded. **p < .01.

Effects of idea generation versus reading on eye parameters are visualized with effect size *r* in Fig. 2. In the idea generation task participants produced more blinks (*Z* = 5.97, *p* < .001, *r* = .61), longer blink duration (*Z* = 4.97, *p* < .001, *r* = .51) and a lower microsaccade count (*Z* = 5.70, *p* < .001, *r* = .58) than in the reading task. But we observed no task difference in the angle of eye vergence (AoEV; *Z* = 0.14, *p* = .890, *r* = .01) and within-trial variability in the AoEV (*Z* = 1.87, *p* = .060, *r* = .19). Pupil diameter was larger during idea generation compared to reading (*Z* = 5.20, *p* < .001, *r* = .53) but within-trial variability of pupil diameter did not differ between task type (*Z* = 0.01, *p* = .999, *r* < .01). More and shorter fixations were made during the idea generation task compared to the reading task (fixation count: *Z* = 5.82, *p* < .001, *r* = .59; fixation duration: *Z* = 5.77, *p* < .001, *r* = .59). Closely associated with the number of fixations, more saccades (*Z* = 5.82, *p* < .001, *r* = .59) and saccades with larger amplitudes (*Z* = 5.40, *p* < .001, *r* = .55) were made during the idea generation compared to the reading task. Like saccade amplitude, microsaccade amplitude was higher during idea generation compared to reading (*Z* = 3.51, *p* < .001, *r* = .36).

In the idea generation task, originality of the two ideas was not correlated with any oculometric parameter (*r*_S_ < .23, *p* > .12).

#### 3.2.2 Background luminance

**Fig. 3:**
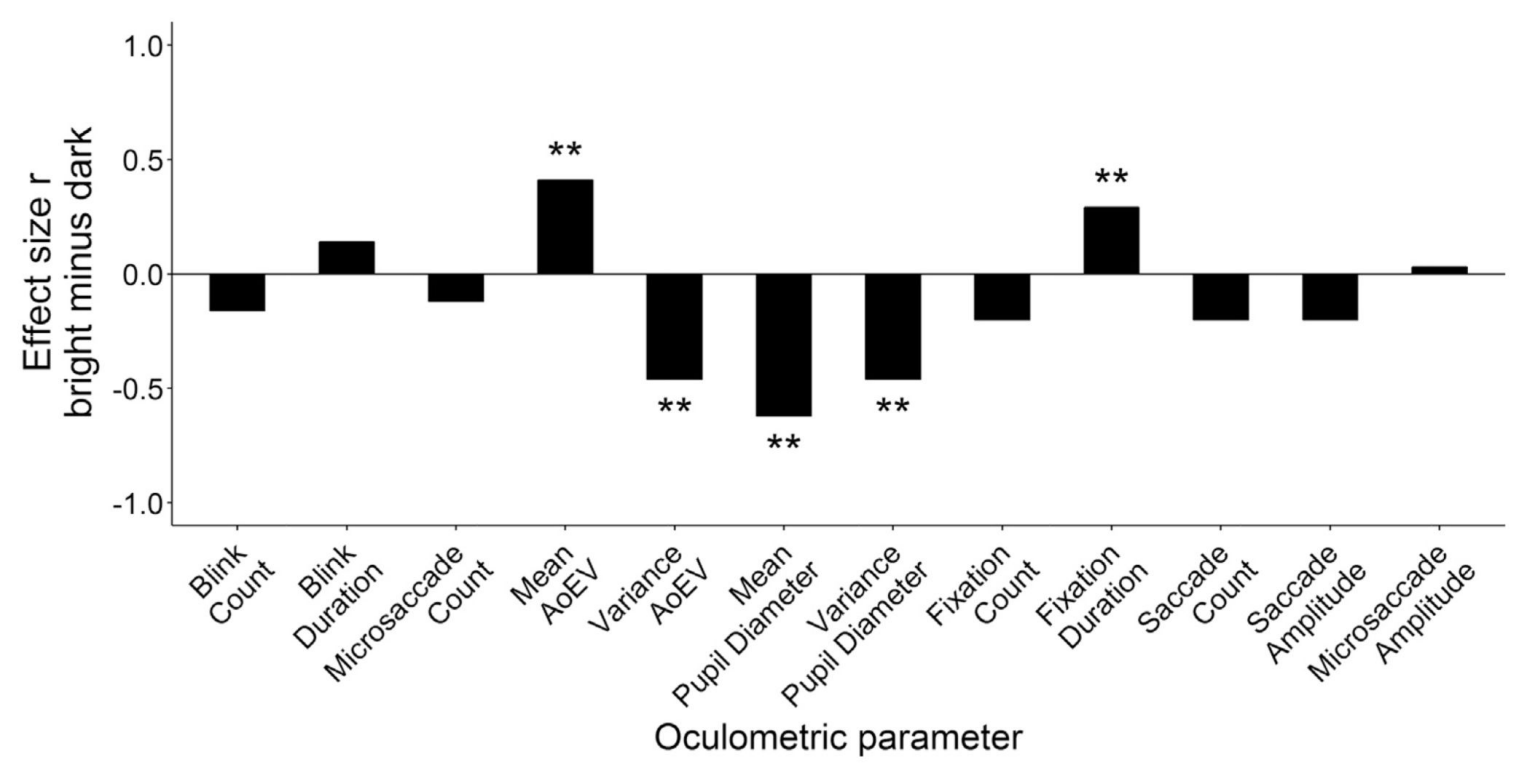
Effects of background luminance on oculometric parameters illustrated by effect size r. Positive effect sizes indicate higher values of the oculometric parameter under bright compared to dark background luminance. **p < .01.

Effect sizes for the influence of background luminance are visualized in Fig. 3. Pupil diameter was smaller with bright background (*Z* = 6.03, *p* < .001, *r* = .62). Angle of eye vergence was larger with bright background compared to the dark background (*Z* = 3.87, *p* < .001, *r* = .41). Both pupil diameter and angle of eye vergence showed less within-trial variability with bright background than with the dark background (pupil: *Z* = 4.48, *p* < .001, *r* = .46, angle of eye vergence: *Z* = 4.47, *p* < .001, *r* = .46). Fixations were longer with bright background than with dark background (*Z* = 2.84, *p* = .004, *r* = .29). Background luminance had no significant effect on fixation count, blink count, blink duration, saccade count, saccade amplitude, microsaccade count and microsaccade amplitude (*Z* < 1.93, *p* > .05, *r* < .28).

## 4 Discussion

The aim of the present study was to examine whether and how characteristics of internally focused cognition are reflected in oculometric parameters. In the following, we discuss our findings with respect to the hypotheses of attenuation of visual input and of coupling of eye behavior to cognitive processes, respectively.

### 4.1 Attenuation of visual input

In support of an active attenuation of visual input through eye behavior during internally focused cognition, participants blinked more often (21 versus 8 blinks per minute) and longer (120 versus 100 ms), and greatly reduced the number of microsaccades (15 versus 4 per minute) during the internal compared to the external task. No effect of attentional direction was found for the mean angle of eye vergence. More and longer blinks induce a longer disruption of the visual input, which may serve as a basic mechanism to reduce the processing of irrelevant external visual information. Salvi et al. (2015) also reported longer blink duration right before insight solutions as compared to analytic solutions. Findings on blink rate have been less consistent for spontaneous internally focused cognition. For example, during mind wandering episodes decreased blink rate was observed only in comparison to a low load task like breath counting (Grandchamp, Braboszcz, & Delorme, 2014) but not in contrast to a higher load task like reading (Uzzaman & Joordens, 2011). Goal-directed internally focused cognition (as in our task of timed idea generation) may require more cognitive resources and therefore a stronger shielding of ongoing internal processes from intruding irrelevant external input than mind wandering.

In further support of attenuation of visual input through eye behavior, participants performed fewer microsaccades during idea generation than during reading (see also Benedek, Stoiser, Walcher, & Körner, 2017). Given the close linkage of microsaccade suppression and perceptual fading (Martinez-Conde et al., 2006), reduced microsaccade activity may favor fading of visual perception and thereby serve as another mechanism to gate out irrelevant external information during internally focused cognition. Given the higher average frequency of microsaccades compared to blinks, microsaccades could be seen as a more time-sensitive indicator of internally focused cognition. Contrary to our exploratory expectations, we found no effect of attention direction on eye vergence. This means that participants mean distance of focus was similar in both tasks.

Overall, the eye behavior pattern of heightened blink rates and reduced microsaccade rates favors the hypotheses of an active attenuation of visual input. In the present study, defocusing was not part of that eye behavior pattern (but see Benedek et al., 2017). Further studies should investigate whether this eye behavior pattern emerges generally during internally focused cognition or if it is specific for certain study conditions. Distractor saliency could be such a critical study condition. People avert gaze more often if a distractor is salient (Doherty-Sneddon & Phelps, 2005). In the present study, external events (appearance of a single letter every 500 ms) during the internal task were perceived as relatively little distracting (1.72 on a scale from 0, *not at all distracting,* to 5, *very distracting*). Some eye behavior patterns related to attenuation of visual input, like eye vergence thus could only become necessary under conditions of higher distractor saliency.

### 4.2 Coupling to internally generated information

Goal-directed internally (vs. externally) focused cognition was associated with larger average pupil diameter. This is in line with recent research on mind wandering (Franklin et al., 2013; Smallwood et al., 2011). But pupil diameter is an intricate eye parameter because it is affected by different variables such as imagined luminance (Laeng & Sulutvedt, 2014) and cognitive load (Piquado et al., 2010), leading to ambiguous results, as demonstrated by Grandchamp et al. (2014) in the domain of mind wandering. While mind wandering seems to be associated with a larger pupil diameter (Franklin et al., 2013; Smallwood et al., 2011), so is increasing cognitive load (Palinko et al., 2010; Piquado et al., 2010). In the present study, idea generation (internally focused cognition) was perceived as more demanding than reading (externally focused cognition). In the internal task, participants had to generate ideas and keep the best two ideas in memory for the remaining time. We tried to account for the working memory load by including some working memory load in our external task. In the external task, participants had to read a letter stream. That also included working memory to a certain point, as participants had to retrieve the previous letters and append the present letter to be able to read the resulting message. Furthermore, they had to remember the whole sentence until the questions appeared. So in both, the external and internal task, participants had to modify material in mind and had to remember something during the trial. Therefore the main difference between the two tasks was the focus of cognition: during the external task participants had to continuously focus on external events (externally focused cognition) and during the internal task they had to focus on internally generated information, namely their ideas and could ignore external events (internally directed cognition). Yet, one cannot fully rule out some differential effects of working memory load on pupil diameter. This point underscores the need to include a wide range of oculometric parameters and to look at the overall pattern instead of only investigating a small subset thereof (e.g. only pupil diameter).

Supporting our hypotheses that eye behavior is linked to the cognitive processes, idea generation and reading differed strongly in their eye behavior. Consistent with the external task’s presentation characteristics (only one letter at a time in the center of the screen), pupil diameter and angle of eye vergence were relatively stable. Moreover, only a few fixations and saccades and only saccades with small amplitudes were made allowing for effective reading of the letter stream. This eye movement pattern was in strong contrast to that of idea generation. Like searching in a real visual environment (Ehrlichman & Micic, 2012), searching for an idea in the mind’s eye evoked a more active eye movement pattern than in reading. Internally focused cognition induced a higher rate of fixations and saccades and a slight but not significant increase in variability of angle of eye vergence. Given that angle of eye vergence can be influenced by imagined changes in depth (McLin & Schor, 1988), those variations could reflect features of imagined ideas during the generation task.

Eye behavior patterns strongly depend on the cognitive processes active and therefore on task characteristics. This implies that the observed differences (e.g., higher fixation counts for internal cognition) may not be intrinsic to internally focused cognition, but can only be interpreted in the context of the different cognitive processes involved in internal and external cognition tasks. It hence is not clear whether task differences in these parameters would also have been observed when tasks had been more similar (e.g., idea generation for a visible object as compared to idea generation for an imagined object; but see Benedek et al., 2017). As a consequence, coupling of eye behavior to cognitive processes may only serve as a reliable indicator of internally versus externally focused cognition when cognitive processes are well understood and differentiable.

### 4.3 Effects of background luminance

Background luminance was varied within tasks in order to test the robustness of attention effects and the sensitivity of parameters to luminance. We observed no interaction between attention direction and luminance supporting the idea that effects of attention direction are robust across different conditions of luminance. As expected, pupil diameter was smaller for bright compared to dark background, and we observed further effects on the mean angle of eye vergence and within-trial variability of pupil diameter and eye vergence. The latter findings may in part be due to eye tracking characteristics itself. Recent studies found a systematic distortion of gaze measurements due to pupil dynamics (Drewes, Zhu, Hu, & Hu, 2014; Eberhardt & Huckauf, 2016).

### 4.4 Implications and future directions

An objective discrimination between internally and externally focused cognition has important implications for cognitive research that often relies on self-report for assessing the focus of attention. Further investigation of the conditions under which attenuation of visual input occurs might help to refine theories of distractor inhibition during internal cognition and of switches between external and internal cognition. For example, load theory (for an overview see Murphy, Groeger, & Greene, 2016) postulates different effects of cognitive and perceptual load on distractor interference during externally focused cognition. Both cognitive and perceptual load also have a strong impact on distractibility during internally focused cognition. Adding to that, Buetti and Lleras (2016) showed that increased cognitive engagement reduces sensitivity to visual events. In a next step, one should investigate if the attenuation of visual input under higher cognitive load and cognitive engagement could be related to changes in the eye behavior pattern (blinks, microsaccade suppression) as found in the present study.

Regarding the properties of the used tasks, one can also interpret the results in the framework of the predictive and reactive control system theory (PARCS, see Tops, Boksem, Quirin, IJzerman, & Koole, 2014). During the reading task, participants had to integrate new incoming visual information (letters) continuously, which would involve mostly the reactive control system or switches between the reactive and predictive control systems of the PARCS. Compared to that, the idea generation task requires a focus on internally generated information and a decoupling from incoming signals, therefore mainly the predictive system should be involved. Attenuation of visual input could indicate the involvement of the predictive control system. Establishing an eye behavior pattern that can indicate this decoupling from the external world reliably not only allows for a more accurate categorization of internally and externally focused cognition between tasks but also within tasks. The latter would be valuable for the investigation of the brain mechanisms associated with switches between the reactive and predictive control systems.

Further, one should investigate individual differences in attenuation and coupling related to eye behavior in the face of distracting visual input. This could bring us closer to the answer why some people are so good at shielding their internal train of thought while others have serious trouble doing so, especially in a world where we are confronted with an immense amount of external stimulation (e.g. adds on webpages, new emails popping up on the desktop).

### 4.5 Conclusions

The present study established that goal-directed internally and externally focused cognition can be reliably discriminated based on eye parameters. Increasing blink rates and durations as well as reduced microsaccade activity may facilitate perceptual attenuation via shut-out and fading of visual information. This eye behavior pattern thus may represent an important mechanism to shield ongoing internal trains of thought from irrelevant, potentially distracting external stimulation, and future research should attempt to test this notion experimentally. Further differences in eye parameters between internally and externally focused cognition may be interpreted in terms of a coupling of differing cognitive demands between idea generation and reading. We conclude that the eyes do not idle during cognitive activities that are independent from sensory information but rather seem to play an active supporting role by shielding internal thought processes as well as reflecting them.

## Figures and Tables

**Table 1:** Median and median absolute deviation (in parentheses) of eye movement variables.

	External Reading task			Internal Alternate Uses Task		
		
	Bright	Dark	Total	Bright	Dark	Total
Pupil diameter (z-transformed)	−0.97 (0.09)	0.73 (0.13)	−0.12 (0.09)	−0.69 (0.12)	0.99 (0.20)	0.14 (0.13)
Pupil diameter variability* (z-transformed)	0.15 (0.06)	0.20 (0.07)	0.18 (0.06)	0.16 (0.05)	0.20 (0.16)	0.17 (0.09)
Angle of eye vergence* (degrees)	7.09 (0.31)	6.83 (0.33)	6.99 (0.25)	7.07 (0.29)	6.91 (0.36)	6.96 (0.22)
Angle of eye vergence variability* (degrees)	0.08 (0.07)	0.14 (0.10)	0.11 (0.09)	0.09 (0.06)	0.15 (0.08)	0.12 (0.08)
Blink count* (N/min)	7.50 (8.53)	7.00 (9.08)	7.88 (8.90)	21.00 (19.64)	20.00 (18.53)	21.19 (20.02)
Blink duration* (ms)	106.03 (41.36)	98.76 (32.69)	100.70 (40.19)	123.59 (36.43)	119.83 (40.38)	121.06 (37.63)
Fixation count (N/min)	35.38 (19.27)	35.00 (19.27)	34.56 (20.11)	59.38 (26.87)	65.75 (26.13)	62.50 (26.04)
Fixation duration* (ms)	1681.78 (1053.46)	1787.90 (1115.02)	1801.53 (1141.41)	947.25 (459.82)	834.44 (408.05)	896.62 (409.96)
Saccade count (N/min)	34.50 (19.27)	34.13 (19.27)	33.63 (20.11)	58.50 (26.69)	65.13 (26.13)	61.69 (26.13)
Saccade amplitude* (degrees of visual angle)	0.52 (0.14)	0.59 (0.25)	0.56 (0.21)	1.17 (0.81)	1.21 (0.74)	1.29 (0.81)
Microsaccade count* (N/min)	13.25 (15.75)	13.00 (16.87)	15.50 (18.63)	3.38 (4.08)	3.25 (4.45)	3.81 (4.73)
Microsaccade amplitude* (degrees of visual angle)	0.38 (0.23)	0.35 (0.20)	0.40 (0.23)	0.49 (0.42)	0.48 (0.38)	0.50 (0.41)

*Note*: Medians of all eye movement variables (median absolute deviation). N = 48.

* Normal distribution violated (significant Shapiro-Wilk test).
